# Ultrastructural perspectives on the enteric nervous system in Parkinson’s disease

**DOI:** 10.3389/fnins.2026.1927448

**Published:** 2026-09-07

**Authors:** Felix Lappe, Jacqueline Heinen-Weiler, Sarah Stahlke, Carsten Theiss

**Affiliations:** 1 Medical Imaging Center (MIC), Faculty of Medicine, Electron Microscopy Medical Analysis – Core Facility (EMMA^CF^), Ruhr University Bochum, Bochum, Germany; 2 Department of Cytology, Faculty of Medicine, Ruhr University Bochum, Bochum, Germany; 3 International Graduate School of Neuroscience (IGSN), Ruhr University Bochum, Bochum, Germany

**Keywords:** electron microscopy, ENS, mitochondria, Parkinson’s disease, ultrastructure

## Abstract

The enteric nervous system (ENS), a central component of the gut-brain axis, is increasingly recognized as an important site in the pathogenesis of neurodegenerative diseases. In Parkinson’s disease (PD) in particular, gastrointestinal dysfunction and pathological changes within the ENS often precede symptoms in the central nervous system (CNS). According to the Braak hypothesis, disease-associated pathology may originate in the gastrointestinal tract and spread to the brain via neural pathways such as the 10*^th^* cranial nerve, the vagus nerve. Consequently, the ENS is considered a potential early site of neurodegenerative processes. Despite this growing interest, most studies investigating ENS pathology rely on fluorescence-based approaches that primarily provide information on protein expression and cellular distribution. While these techniques have advanced our understanding of enteric neuronal networks, they offer limited insight into subcellular organization. Ultrastructural analysis of the CNS has already identified characteristic changes at the subcellular level, including morphological alterations of synapses, changes in the endolysosomal system, and mitochondrial dysfunction. Mitochondrial dysfunction represents a key mechanism in PD, as demonstrated in toxin models such as rotenone exposure and in genetic forms involving mutations in PINK1 or PRKN. However, comparable ultrastructural investigations of the ENS remain scarce. This review highlights the importance of ultrastructural investigations of the ENS for understanding early neurodegenerative processes and discusses how electron microscopy (EM) may reveal previously underappreciated cellular and subcellular alterations in enteric neurons. Particular attention is given to mitochondrial pathology and the need for systematic ultrastructural analyses of the ENS in models and human studies of PD.

## 1 Introduction

The ENS was already extensively studied using EM in the 1970s and 1980s, with particular emphasis on the ultrastructural organization of enteric neurons and glial cells (Baumgarten et al., 1970; Gabella, 1972; Cook and Burnstock, 1976a; Wilson et al., 1981). These early studies provided an initial understanding of the cellular architecture of the ENS, but they were limited to healthy tissue from animal models. Since then, electron microscopic studies of the ENS have been rare.

Nevertheless, scientific and societal interest in the ENS has grown significantly in recent years. The gastrointestinal tract is no longer viewed exclusively as a digestive organ but increasingly as a complex system with multifaceted functions. In addition to metabolism and immune regulation, neural signal processing also plays a central role (Gershon, 1999; Furness, 2012). Not least due to the close bidirectional communication between the ENS and the CNS within the context of the gut-brain axis, the ENS is increasingly coming into focus (Carabotti et al., 2015). This communication is based on a complex interplay of hormonal, immunological, metabolic, and neuronal signaling pathways, with direct connections, such as the vagus nerve, enabling interaction between the gut and the brain (Forsythe et al., 2014; Cryan et al., 2019). Thus, the ENS represents a potential interface between environmental factors and the CNS and is increasingly being discussed as a possible point of origin for neurodegenerative diseases. In PD, several models have been proposed to explain the relationship between peripheral and central pathology (Borghammer and Van Den Berge, 2019). The “brain-first” model suggests that pathological processes may initially originate within the CNS, with peripheral structures such as the ENS becoming affected secondarily during disease progression (Borghammer and Van Den Berge, 2019; Horsager et al., 2020). In this concept, neurodegenerative changes are thought to arise primarily within central neuronal circuits, and subsequent involvement of the autonomic nervous system and peripheral tissues may occur as part of a broader disease process (Borghammer and Van Den Berge, 2019; Horsager et al., 2020). In contrast, the “body-first” model proposes that pathology may initially arise in peripheral tissues, including the ENS and peripheral autonomic nervous system, and subsequently spread to the CNS through interconnected pathways (Hawkes et al., 2007; Borghammer and Van Den Berge, 2019). These models highlight the heterogeneity of PD and suggest that the ENS may either represent an early site of disease initiation or a structure affected during systemic disease. The Braak hypothesis posits an early manifestation of PD pathology in the ENS and suggests a possible spread via the vagus nerve as a conduit (Braak et al., 2003b). In the context of PD, numerous studies have detected pathological deposits of α-synuclein, in some cases as early as the initial stages of the disease (Lebouvier et al., 2010; Shannon et al., 2012; Hilton et al., 2014; Ohlsson and Englund, 2019). Experimental studies also provide evidence of a possible spread of pathological aggregates from the gut to the brain along neural pathways (Holmqvist et al., 2014). Despite these rapid advances in research on the gut-brain axis, most current studies on the structure and function of the ENS rely on light microscopy and fluorescence-based methods. While these techniques provide valuable information on protein expression, cellular morphology, three-dimensional organization and functional activity of neuronal networks they remain limited in their ability to resolve the ultrastructural organization of subcellular compartments at nanometer resolution (Desmet et al., 2017; Hazart et al., 2025, 2026). In contrast, ultrastructural analysis of the CNS has already made crucial contributions to our understanding of neurodegenerative diseases, particularly regarding synaptic changes, endolysosomal involvement, and mitochondrial dysfunction (Trimmer et al., 2000; Baloyannis et al., 2006; Boassa et al., 2013; Shahmoradian et al., 2019). Against this backdrop, it is noteworthy that comparable ultrastructural studies of the ENS, particularly under pathological conditions, have been largely absent to date.

The aim of this review is therefore to highlight the significance of ultrastructural analyses of the ENS in the context of neurodegenerative diseases and to demonstrate the potential of EM for understanding pathological changes in the ENS. Particular focus is placed on mitochondrial changes as a central mechanism of neurodegenerative processes.

## 2 The enteric nervous system and its role in neurodegeneration

**FIGURE 1:**
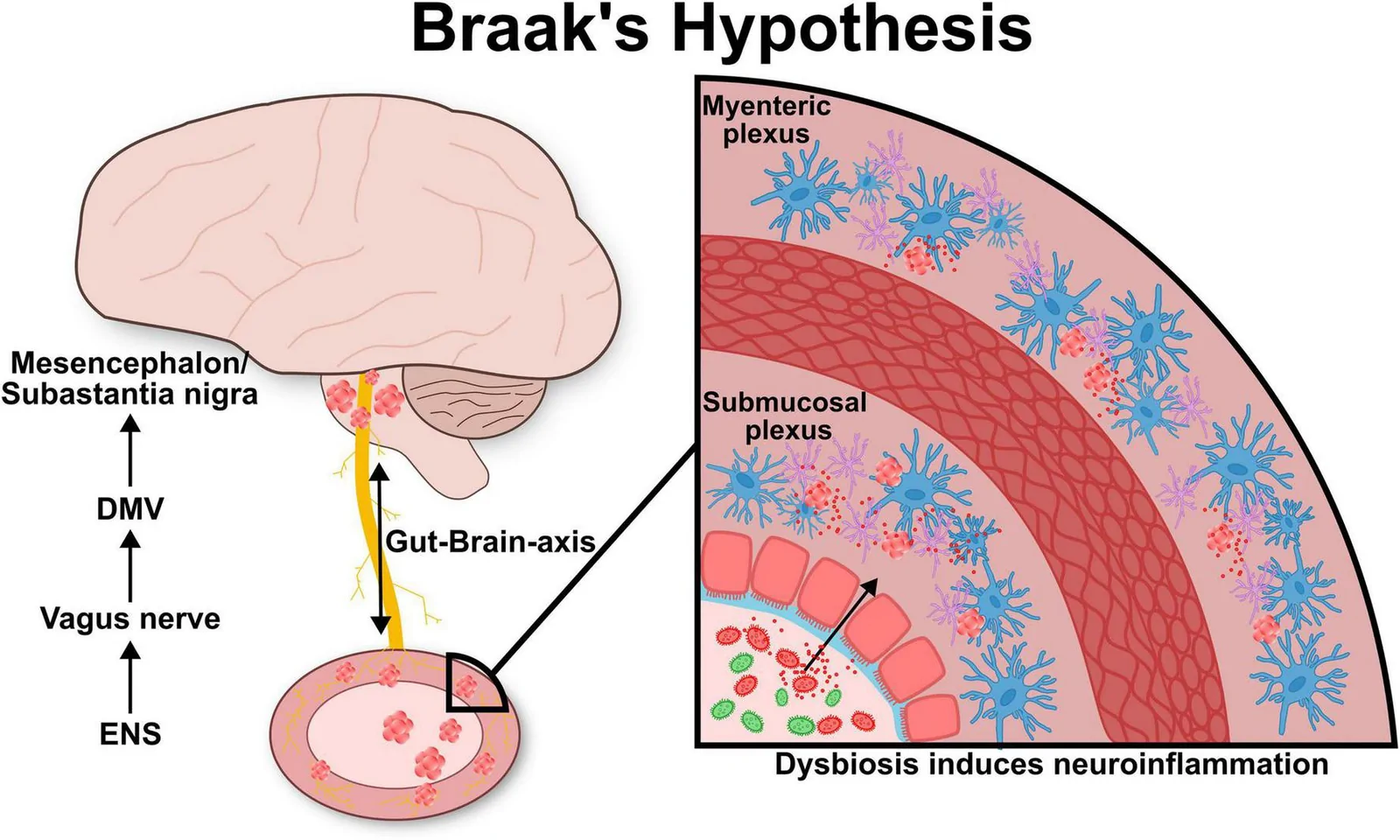
llustration of the gut-brain axis and the Braak hypothesis as a possible model of Parkinson’s disease (PD). Dysbiosis and intestinal barrier dysfunction may promote α-synuclein (red aggregates) misfolding within the enteric nervous system (ENS). The Figure depicts the proposed signaling pathway via the vagus nerve, the dorsal motor nucleus of the vagus (DMV), and subsequently to the mesencephalon/substantia nigra. Enteric neurons (blue), glial cells (violet), microbial and inflammatory signals (red dots) within the intestinal lumen are indicated.

In recent years, the ENS has increasingly been recognized as a potentially early and previously underestimated component in the pathogenesis of neurodegenerative diseases. Especially in the context of PD, there is growing evidence that pathological changes in the gastrointestinal tract may precede the onset of classic motor symptoms (Shannon et al., 2012; Hilton et al., 2014). Clinical observations show that, in addition to classic symptoms such as tremor, rigidity, and bradykinesia, gastrointestinal complaints such as constipation often occur years to decades before diagnosis, which may indicate early involvement of the ENS (Abbott et al., 2001; Jankovic, 2008; Postuma and Berg, 2019). In this context, the Braak hypothesis provides a central explanatory framework. Braak et al. (2003a,b) postulate an early manifestation of pathology in peripheral tissues, particularly in the ENS, possibly triggered by a pathogen capable of overcoming the epithelial barrier. From there, the hypothesis posits a spread along neural connections into the CNS (Figure 1; Braak et al., 2003a,b).

However, PD is increasingly recognized as a heterogeneous disorder, and alternative models of disease initiation, including body-first and brain-first concepts, suggest that pathological processes may originate either in peripheral tissues or within the CNS itself (Borghammer and Van Den Berge, 2019; Horsager et al., 2020). The structural and functional organization of the ENS is of central importance for evaluating this hypothesis. The ENS is a highly differentiated neural network extending along the entire gastrointestinal tract, from the esophagus to the anal canal, and, with an estimated 200–600 million neurons, constitutes one of the largest neural networks in the human body (Gershon, 1999; Furness, 2006, 2012; Michel et al., 2022). Functionally, it plays a central role in the regulation of gastrointestinal processes by coordinating autonomic reflexes that control motility, secretion, and absorption, and is essential for maintaining the intestinal barrier and modulating immunological processes (Savidge et al., 2007; Bach-Ngohou et al., 2010; Brown et al., 2016; Progatzky et al., 2021; Sharkey and Mawe, 2023). Beyond its intrinsic organization, the ENS is in continuous interaction with the intestinal microbiome and therefore represents a central component of the gut-brain axis (Cryan et al., 2019). Microbial metabolites and microbiota-driven immune signaling contribute to the regulation of intestinal homeostasis and can influence ENS function as well as intestinal barrier integrity (Cryan et al., 2019; Morais et al., 2021). Increasing evidence suggests that alterations in the gut microbiome (dysbiosis) promote chronic inflammation, oxidative stress, and disturbances of intestinal homeostasis, all of which have been implicated in PD (Zhu et al., 2022). Microbiome-derived factors may therefore represent additional modulators of ENS integrity by influencing cellular stress pathways, mitochondrial homeostasis, and protein quality control mechanisms (Gabrielli et al., 2024; Guo et al., 2025). These processes may ultimately manifest at the subcellular level, including changes in mitochondrial morphology, organelle remodeling, synaptic changes, and potentially altered α-synuclein homeostasis. However, whether microbiome-associated alterations are directly reflected by characteristic ultrastructural changes within the ENS remains largely unknown. Addressing this question using ultrastructural approaches may provide new insights into how environmental factors contribute to early cellular changes in PD-associated neurodegeneration. These diverse interactions highlight the central role of the ENS within the gut-brain axis. A distinctive feature of the ENS is its capacity for largely autonomous reflex processing, which can occur independently of the CNS, although bidirectional communication with the CNS is maintained, particularly via the vagus nerve (Bayliss and Starling, 1899, Forsythe et al., 2014; Bonaz et al., 2018; Cryan et al., 2019). Anatomically, the ENS is divided into two main plexuses: the myenteric plexus (Auerbach’s plexus), which is located between the longitudinal and circular muscles and extends throughout the gastrointestinal tract from the esophagus to the internal anal sphincter, and the submucosal plexus (Meissner’s plexus), which is located in the submucosa and is more pronounced in the small and large intestines (Gershon, 1999; Furness, 2006; Sharkey and Mawe, 2023). Both plexuses consist of interconnected ganglia and form a dense, functionally integrated neural network (Furness, 2006). At the cellular level, the ENS is composed of a multitude of specialized neurons and enteric glial cells. The enteric neurons exhibit marked functional and neurochemical diversity and can be subdivided into motor neurons, interneurons, and primary afferent neurons, which together form complex neural circuits for the coordination of gastrointestinal functions (Costa et al., 2000; Fung and Van den Berghe, 2020). This functional diversity is reflected in central neurotransmitter systems, including, for example, acetylcholine as the most important excitatory transmitter (Fahrenkrug et al., 1978; Costa et al., 1992, 2000). In addition, other neuromodulators such as ATP or serotonin (5-HT) contribute to the fine-tuning of enteric signaling processes (Holzer and Holzer-Petsche, 1997; Monro et al., 2004). This complex neurochemical organization enables precise regulation of gastrointestinal functions. However, the functional complexity of the ENS is not determined solely by enteric neurons but is significantly complemented by the equally highly differentiated enteric glial cells, which were long considered a purely supportive cell type. Enteric glial cells represent an essential and long-underestimated cell type of the ENS. They form a dense network that surrounds enteric neurons and nerve fibers (Gabella, 1972). Functionally, however, their roles extend far beyond a purely supportive function (Gulbransen and Sharkey, 2012). Enteric glial cells influence neuronal activity, regulate oxidative stress, and are involved in neurogenesis in the ENS (Abdo et al., 2010; Laranjeira et al., 2011; McClain et al., 2015). Furthermore, they play a central role in interacting with the immune system through the release of immunomodulatory factors (Brown et al., 2016; Progatzky et al., 2021). They are also increasingly recognized as playing a significant role in maintaining the epithelial barrier (Savidge et al., 2007; Bach-Ngohou et al., 2010). Due to these functions, glial cells could potentially become key players in neuroinflammatory and neurodegenerative processes in the gastrointestinal tract.

## 3 Ultrastructural organization and neurodegenerative changes in the ENS

Against this backdrop, the ENS, with its complex interplay of neurons, glial cells, immune cells, and epithelial structures, is increasingly becoming the focus of research. The close functional interconnection of these components suggests that even subtle changes at the cellular and subcellular levels can contribute significantly to the onset and progression of gastrointestinal and neurodegenerative processes. High-resolution imaging techniques are of central importance for precisely characterizing such structural changes.

Ultrastructural analyses have already provided crucial insights into the cellular and subcellular mechanisms of neurodegenerative diseases in the CNS. EM allows the visualization of organelles, synaptic structures, and intracellular compartments at high-resolution, thereby enabling the early identification of pathological changes that may precede cell death. In comparison, the ENS has so far been systematically studied at the ultrastructural level only to a limited extent.

At the ultrastructural level, enteric neurons and glial cells exhibit characteristic differences that reflect their respective functional roles. Neurons possess a large, usually eccentrically located nucleus with a prominent nucleolus, as well as cytoplasm containing abundant rough endoplasmic reticulum (rER), mitochondria, and cytoskeletal structures (Baumgarten et al., 1970; Gabella, 1972). Particularly striking is the distribution of mitochondria along the neurites and presynaptic regions, where they play a central role in supplying energy to synaptic processes (Baumgarten et al., 1970; Cook and Burnstock, 1976a). Another characteristic feature of the ENS are axonal varicosities, which contain synaptic vesicles and serve as functional sites of release for neuronal signals (Gabella, 1972; Cook and Burnstock, 1976a). In contrast, enteric glial cells have smaller cell bodies, denser nuclei, and a lower organelle density; however, they are rich in intermediate filaments and exhibit a close structural and functional association with neuronal elements (Gabella, 1972). This specific ultrastructural organization forms the structural basis for the functional properties of the ENS and is also the level at which early pathological changes become apparent.

**FIGURE 2:**
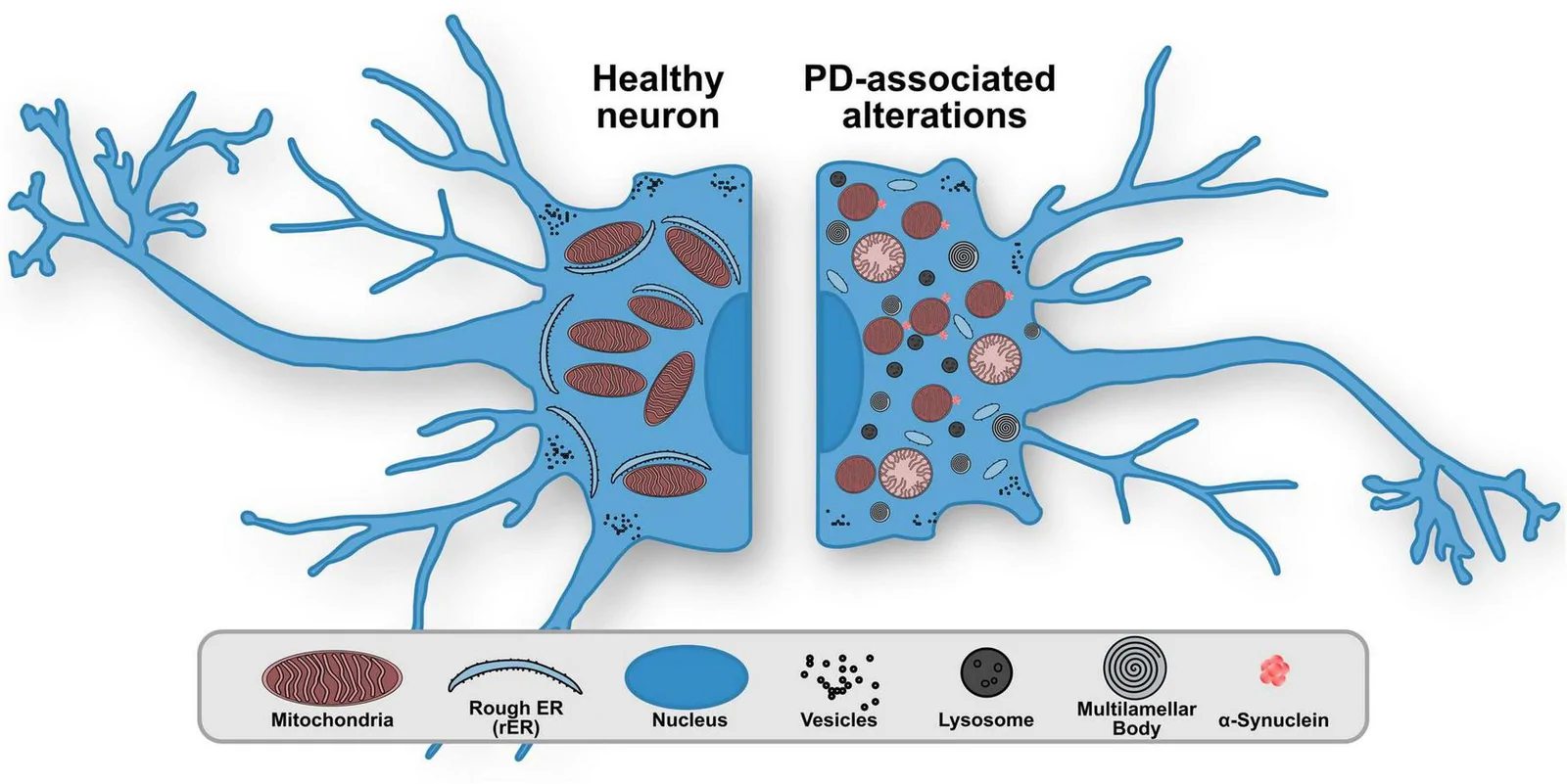
Schematic comparison of a healthy and Parkinson’s disease (PD)-associated neuron. The illustrated enteric neuron highlights characteristic ultrastructural alterations associated with PD extrapolated from studies in the central nervous system. Hallmarks include mitochondrial fragmentation and swelling, cristae disorganization, endoplasmic reticulum dilatation, accumulation of autophagic and lysosomal structures, reduced synaptic vesicle density and altered neurite morphology.

While neurodegenerative mechanisms in the ENS have hardly been studied at the ultrastructural level to date, there are already numerous studies of the CNS that provide a potential frame of reference for the ENS. In neurodegenerative processes in the CNS, synaptic structures, among other things, are typically affected early on. In PD, ultrastructural studies of the CNS reveal a reduction in dendritic spines as well as a significantly reduced branching of the dendritic tree (Baloyannis et al., 2006). Additionally, changes in the density and morphology of synaptic vesicles can be observed (Baloyannis et al., 2006). The organelles in the synaptic regions are also affected: the smooth endoplasmic reticulum (sER) appears vacuolated, and mitochondria appear smaller and more rounded (Figure 2; Baloyannis et al., 2006). Such changes can significantly impair synaptic transmission and are considered early events in the course of the disease. Merino-Galán et al. (2025) demonstrated possible compensatory adaptations of the sER in a premotor Parkinson’s model. Such a precise measurement of the increase in sER surface area was only possible through systematic EM analysis. EM analyses have also contributed significantly to our understanding of the role of the endolysosomal system in PD within the CNS. An accumulation of autophagic vesicles as well as changes in lysosomal structures has been described in degenerating neurons of the substantia nigra (Stefanis et al., 2001). The injection of α-synuclein preformed fibrils (PFFs) into the substantia nigra of mice also induced an accumulation of large lysosomal structures (Geibl et al., 2024). Terminal lysosomes exhibited a multilamellar appearance (Geibl et al., 2024). In a DJ-1 knockout model, these multilamellar structures were quantitatively analyzed and found to be significantly increased in the Parkinson’s model (Krebiehl et al., 2010). An accumulation of these structures could also represent a central aspect of dopaminergic neuron degeneration. These findings suggest a central role for disrupted endolysosomal processes in the pathogenesis. Additionally, it has been shown that α-synuclein is directly associated with multivesicular bodies, lysosomes, and presynaptic endomembrane systems and influences their organization (Trimmer et al., 2000). Impaired lysosomal function not only promotes the accumulation of α-synuclein but is itself further exacerbated by α-synuclein aggregates, thereby facilitating a pathological feedback mechanism (Boassa et al., 2013). In addition to synaptic and endolysosomal alterations, mitochondrial dysfunction represents another central pathomechanism. Ultrastructural studies of human postmortem brains, as well as various animal and cell models, have identified morphological mitochondrial alterations. Characteristic changes include swelling, vacuolization, fragmentation, and a disorganized and dilated cristae structure, which are associated with functional deficits in the respiratory chain (Lach et al., 1992; Trimmer et al., 2000; Stichel et al., 2007; Zhang et al., 2017; Ganjam et al., 2019; Slezia et al., 2023; Geibl et al., 2024). Garcia-Caballero et al. (2026) demonstrated that mitochondrial changes can and must be not only qualitatively described using EM but also quantitatively verified, using an animal model involving manganese inhalation. Mangenese exposure is used to model Parkinsonism because it induces mitochondrial dysfunction, oxidative stress and selective neurodegeneration, thereby reproducing several key pathological mechanisms associated with PD (Ordoñez-Librado et al., 2010). Here, an increase in mitochondrial number accompanied by a decrease in mitochondrial surface area, as well as disorganization of the cristae, in the Parkinson’s model were described. These findings indicate that mitochondrial dysfunction is by no means merely a concomitant feature, but rather a central driver of neurodegenerative processes. Therefore, ultrastructural analysis of mitochondria is an essential component of research into neurodegenerative diseases (Table 1).

**TABLE 1:** Ultrastructural hallmarks in central nervous system and expected in enteric nervous system.

References	Species/model	Disease	Method	Main ultrastructural finding in CNS	Potential relevance for ENS
Baloyannis et al., 2006	Human postmortem cortex	Parkinson’s disease	TEM	Loss of dendritic spines and reduced dendritic arborization; decreased synaptic vesicle density; vacuolated smooth ER; small, rounded mitochondria	Similar synaptic and organellar abnormalities may affect enteric neurons
Merino-Galán et al., 2025	Rat rotenone model (premotor PD)	Parkinson’s disease	TEM	Significant increase in smooth ER surface area and ER complexity in nigral neurons, interpreted as adaptive remodeling	ER remodeling may represent an early response in enteric neurons
Stefanis et al., 2001	PC12 cells expressing A53T α-synuclein	Parkinson’s disease	TEM	Accumulation of autophagic vacuoles and autophagosome-like structures associated with α-synuclein toxicity	Similar defects in autophagy could contribute to α-synuclein pathology in the ENS
Boassa et al., 2013	Primary neurons from transgenic mice	Parkinson’s disease	Immuno-EM	α-Synuclein localized to multivesicular bodies, lysosomes and presynaptic membranes; altered endomembrane organization	Comparable alterations could influence α-synuclein trafficking in enteric neurons
Krebiehl et al., 2010	DJ-1 knockout mouse	Parkinson’s disease	TEM	Increased numbers of multilamellar bodies and abnormal mitochondrial morphology	Lysosomal and mitochondrial pathology may also occur in the ENS
Geibl et al., 2024	Mouse α-synuclein PFF model	Parkinson’s disease	TEM	Enlarged terminal lysosomes with multilamellar appearance; disrupted mitochondrial cristae	Similar endolysosomal pathology may occur in enteric α-synucleinopathy
Lach et al., 1992	Human substantia nigra	Parkinson’s disease	TEM	Swollen mitochondria with disrupted cristae in degenerating neurons	Mitochondrial pathology may represent a conserved mechanism
Trimmer et al., 2000	Cybrid cell model	Parkinson’s disease	TEM	Mitochondria showed swelling, vacuolization and loss of cristae architecture	Similar mitochondrial alterations could affect enteric neurons
Stichel et al., 2007	A30P α-synuclein transgenic mouse	Parkinson’s disease	TEM	Degenerating mitochondria with fragmented and dilated cristae accompanied by axonal pathology	Mitochondrial degeneration may precede enteric neurodegeneration
Zhang et al., 2017	MPTP mouse model	Parkinson’s disease	TEM	Fragmented mitochondria and abnormal cristae structure associated with impaired mitophagy	Defective mitochondrial turnover may also contribute to ENS pathology
Ganjam et al., 2019	Human dopaminergic neurons	Parkinson’s disease	TEM and tomography	α-Synuclein caused mitochondrial fragmentation and altered inner membrane architecture	Similar mechanisms may operate in enteric neurons containing α-synuclein aggregates
Slezia et al., 2023	6-OHDA mouse model	Parkinson’s disease	TEM	Early mitochondrial abnormalities with swelling and disrupted cristae before overt neuronal loss	Comparable early ultrastructural changes might be detectable in the ENS
Garcia-Caballero et al., 2026	Mouse manganese inhalation model	Parkinsonism	TEM + quantitative morphometry	Increased mitochondrial number, reduced mitochondrial area and severe cristae disorganization	Quantitative EM approaches could be transferred to ENS disease models

## 4 From mitochondrial dysfunction to neurodegeneration: mechanisms of cellular destabilization

Mitochondria perform key functions in neuronal homeostasis, including ATP synthesis, regulation of calcium homeostasis, and control of oxidative stress responses (Moon and Paek, 2015). At the subcellular level, however, the interaction between mitochondria and the endoplasmic reticulum (ER) also represents an important mechanism for maintaining neuronal homeostasis. These mitochondria-ER contacts (mitochondria-associated membranes, MAMs) are particularly important for the regulation of calcium signaling, lipid transfer, and cellular stress responses, and thus contribute significantly to the maintenance of cellular balance (van Vliet and Agostinis, 2018). Recent studies show that these interactions are also detectable in the ENS and may be altered in neurodegenerative models (Delfino et al., 2024). It is therefore not surprising that mitochondrial dysfunction also plays a key role in PD. Various triggers are directly linked to characteristic pathological changes and clinical symptoms. In addition to age as a significant risk factor, environmental and genetic factors also play an important role (Moon and Paek, 2015; Amorim et al., 2022). Toxins such as rotenone and/or 1-methyl-4-phenyl-1,2,3,6-tetrahydropyridine (MPTP) also lead to disrupted electron transport by inhibiting Complex I of the mitochondrial respiratory chain, which is associated with reduced ATP synthesis and simultaneously contributes to increased formation of reactive oxygen species (ROS) (Palmer et al., 1968; Nicklas et al., 1987; Ali et al., 1994; Sherer et al., 2003). The increased ROS production triggers oxidative stress, which damages cellular structures such as proteins, lipids, and DNA; in the long term, this can promote neuronal dysfunction and neurodegenerative processes in the ENS (Alam et al., 1997; Jenner, 2003). Due to their high energy requirements and limited capacity for regeneration, neurons are particularly susceptible to disruptions in mitochondrial function, underscoring their role in the pathogenesis of neurodegenerative diseases (Mattson et al., 2008).

Importantly, mitochondrial dysfunction should not be considered as an isolated pathological event but rather as part of a complex network of interacting mechanisms involving α-synuclein accumulation and lysosomal dysfunction. Increased oxidative stress and impaired mitochondrial homeostasis can promote protein misfolding and aggregation of α-synuclein, whereas pathological α-synuclein species may further impair mitochondrial integrity, energy metabolism, and intracellular trafficking (Lin et al., 2019). At the same time, defects in lysosomal degradation pathways can reduce the clearance of misfolded proteins and organelles and thereby facilitate the accumulation and persistence of α-synuclein aggregates and damaged mitochondria (Lin et al., 2019). These reciprocal interactions may establish a self-amplifying cycle of cellular stress, in which mitochondrial dysfunction, impaired protein homeostasis, and defective degradation pathways progressively contribute to neuronal destabilization and degeneration within the ENS.

**FIGURE 3:**
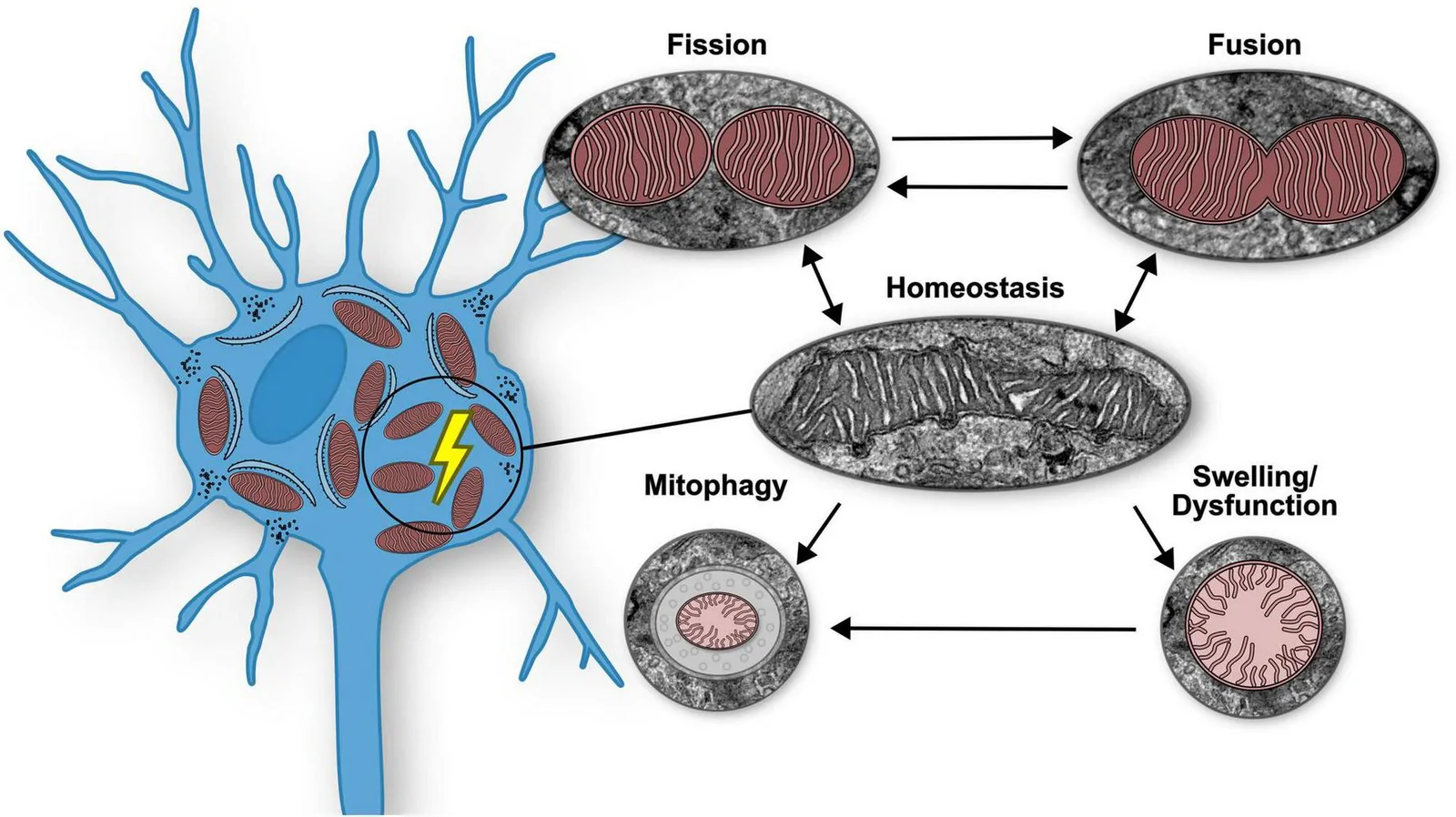
Schematic overview of mitochondrial homeostasis and pathological alterations in Parkinson’s disease (PD). The Figure illustrates mitochondrial fission, fusion, mitophagy, and mitochondrial swelling and dysfunction as key responses and alterations associated with disrupted mitochondrial homeostasis.

In addition to this direct impairment of mitochondrial function, mitochondrial biogenesis and the interplay of fusion and fission processes also appear to play a central role in PD. These processes are essential for adapting to metabolic demands as well as for the distribution and renewal of mitochondrial networks within the cell (Westermann, 2010). Disrupted dynamics and an imbalance between fusion and fission are increasingly being linked to PD (Figure 3).

Rappold et al. (2014) demonstrated that inhibition of the mitochondrial GTPase dynamin-related protein-1 (Drp1), a key fission-associated protein, exerts neuroprotective effects in various animal models and partially restores deficits in dopamine release. The maintenance of mitochondrial function relies on tightly regulated quality control mechanisms that remove damaged organelles and preserve mitochondrial integrity. Hereditary Parkinson’s syndromes involving mutations in the PINK1 and Parkin proteins, which, under normal conditions, are key components of mitophagy, result in damaged mitochondria no longer being efficiently degraded (Kitada et al., 1998; Valente et al., 2004; Narendra et al., 2008, 2010). This leads to the accumulation of defective mitochondria in the cell, thereby promoting neuronal death (Narendra et al., 2008). Overall, numerous genetic risk factors have been identified and are collectively referred to as PARK genes (Klein and Westenberger, 2012). However, only a subset of these genes, including PINK1, PRKN and DJ-1, directly regulates mitochondrial homeostasis, mitophagy or oxidative stress responses (Pickrell and Youle, 2015). In this context, mitochondrial stress responses play a decisive role in determining the outcome between cell survival and cell death. In addition to apoptosis, mediated by mitochondrial outer membrane permeabilization (MOMP), the opening of the mitochondrial permeability transition pore (mPTP) also plays a central role (Halestrap, 2009; Kalkavan and Green, 2018). While MOMP leads to the release of pro-apoptotic factors such as cytochrome c, the opening of the mPTP can result in a loss of mitochondrial membrane potential, massive ATP depletion, and ultimately cell death (Halestrap, 2009; Bernardi et al., 2015; Kwong and Molkentin, 2015; Salvador-Gallego et al., 2016). Both mechanisms represent critical switching points between cellular adaptation and irreversible degeneration. A key aspect of the significance of mitochondrial dysfunction lies in the vulnerability of specific neuronal populations. Dopaminergic neurons are especially susceptible to mitochondrial dysfunction due to a combination of several factors: These include high energy demands, extensive axonal arborization, and continuous calcium loading resulting from autonomic pacemaker activity (Bonci et al., 1998; Guzman et al., 2009; Matsuda et al., 2009; Pacelli et al., 2015). All of this increases the metabolic burden on the mitochondria and heightens their vulnerability to oxidative stress. Changes in mitochondria, synapses, or the cytoskeleton could provide significant clues to early pathological processes. Although these mechanisms have been primarily established for dopaminergic neurons in the CNS, mitochondrial dysfunction is likely to be relevant for the ENS more broadly. Enteric neurons, irrespective of their neurotransmitter phenotpye, also exhibit high metabolic demands and rely on intact mitochondrial function to sustain axonal transport, neurotransmission and synaptic maintenance. However, whether different enteric neuronal subtypes display differential susceptibility to mitochondrial dysfunction remains largely unknown and represents an important area for future investigation.

## 5 Electron microscopy methods: developments and limitations in the context of the enteric nervous system

Methodologically, ENS alterations are investigated using complementary imaging techniques with different spatial resolutions and levels of information. Conventional fluorescence microscopy enables visualization of neuronal and glial populations as well as disease-associated markers such as α-synuclein within intestinal plexus structures (Furness, 2006; Drolet et al., 2009; Shannon et al., 2012; Hazart et al., 2025). A few studies have expanded this scope by specifically investigating mitochondrial parameters of the ENS using fluorescence-based methods. By combining calcium imaging with analyses of the mitochondrial membrane potential, Desmet et al. (2017) were able to investigate the activity of enteric neurons in living duodenal biopsies from Parkinson’s patients for the first time. However, no significant differences in neuronal activity or mitochondrial function were observed in the submucosal plexus compared to control subjects (Desmet et al., 2017). In contrast, Baumuratov et al. (2016) demonstrated, based on fluorescence microscopy analyses of colon biopsies, marked changes in mitochondrial morphology in enteric neurons of Parkinson’s patients. The mitochondria exhibited increased density and total mass, accompanied by a reduction in individual size (Baumuratov et al., 2016). This suggests a possible compensatory adaptation or early mitochondrial dysfunction in the ENS. The major limitation of fluorescence microscopy is its insufficient resolution for detailed analysis of subcellular structures such as membranes, organelles, and macromolecular complexes at nanometer scale (Huang et al., 2009; Sahl et al., 2017).

**FIGURE 4:**
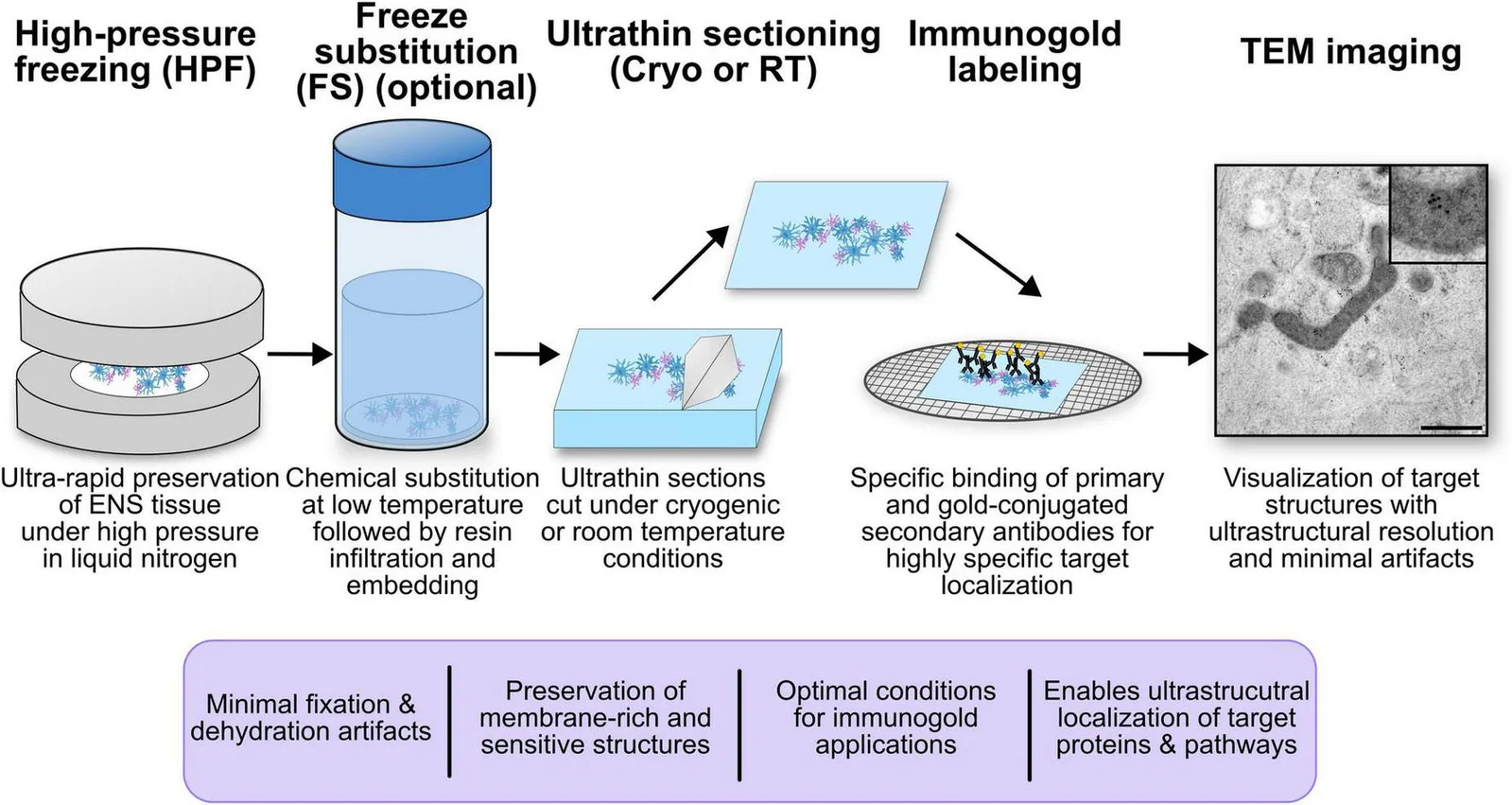
Schematic overview of the workflow combining high-pressure freezing (HPF) with immunogold labeling for transmission electron microscopy (TEM). Following ultra-rapid cryofixation samples undergo optional freeze substitution (FS), rein infiltration and embedding. Ultrathin sections are prepared and subsequently immunolabeled using gold-conjugated secondary antibodies for ultrastructural localization of target proteins. Scale Bar: 0.5 μm.

Electron microscopic analyses of the ENS are based primarily on two complementary techniques: transmission electron microscopy (TEM) and scanning electron microscopy (SEM) (Baumgarten et al., 1970; Gabella, 1972; Cook and Burnstock, 1976a; Wilson et al., 1981; Böttner et al., 2015; Nakanishi et al., 2020). TEM enables analysis of intracellular compartments, synapses, organelles, and other subcellular structures, whereas SEM provides complementary information on surface morphology and tissue architecture (Williams and Carter, 2009; Knott and Genoud, 2013, Kizilyaprak et al., 2019). Both methods are used in ENS research but address different levels of biological organization. Correlative light and electron microscopy (CLEM) combines fluorescent labeling with ultrastructural resolution, allowing molecular signals to be correlated with their subcellular localization (Begemann and Galic, 2016; van den Dries et al., 2022). This allows functional processes such as protein aggregation, synaptic activity, or glial responses within enteric plexus structures to be directly correlated with their ultrastructural localization. In the ENS, for example, CLEM was used to correlate the subcellular distribution of α-synuclein with synaptic and membrane-associated compartments and to structurally localize and identify early pathological aggregation processes (Shahmoradian et al., 2019). Furthermore, Lewy bodies contain not only of fibrillar α-synuclein but also membrane-bound organelles, including vesicles, lysosome-like structures, autophagosomal compartments, and dysmorphic mitochondria (Shahmoradian et al., 2019). Using CLEM, the group led by Choi et al. (2022) was able to demonstrate that mitochondria also play a central role in the aggregation of α-synuclein. Mitochondrial membranes, particularly cardiolipin, appear to be a trigger for the oligomerization of α-synuclein (Choi et al., 2022). Pre- and post-embedding approaches differ mainly in antibody accessibility and ultrastructural preservation (Jones, 2016). Closely related to these approaches are tissue preparation methods that aim to minimize structural artifacts caused by conventional chemical fixation while preserving antigen accessibility for immunogold-based detection methods. The Tokuyasu method is well-suited for immunogold labeling though gentle fixation and cryogenic ultrathin sectioning (Tokuyasu, 1973). It offers excellent antigen accessibility and is therefore frequently used for immunogold localization; however, compared with fully cryogenic methods, it results in poorer ultrastructural preservation (Cortese et al., 2009; Mobius, 2009). Fully cryogenic approaches, such as high-pressure freezing (HPF), enable ultra-rapid preservation of the ENS architecture while minimizing fixation- and dehydration-associated artifacts (Studer et al., 2008; Mobius, 2009). Frozen samples can subsequently be processed by freeze substitution (FS) and resin embedding or directly sectioned into ultrathin sections under cryogenic conditions and analyzed using cryo-TEM (Studer et al., 2008; Chlanda and Sachse, 2014). In particular, membrane-rich and sensitive subcellular structures can be visualized with significantly fewer artifacts using this method (Studer et al., 2008). Furthermore, this method creates optimal conditions for immunogold applications (Figure 4). Particularly in the CNS, researchers have already recognized the opportunity to localize target structures with ultrastructural resolution, thereby potentially deepening our understanding of various signaling pathways (van Lookeren Campagne et al., 1991; Petralia and Wang, 2021).

**FIGURE 5:**
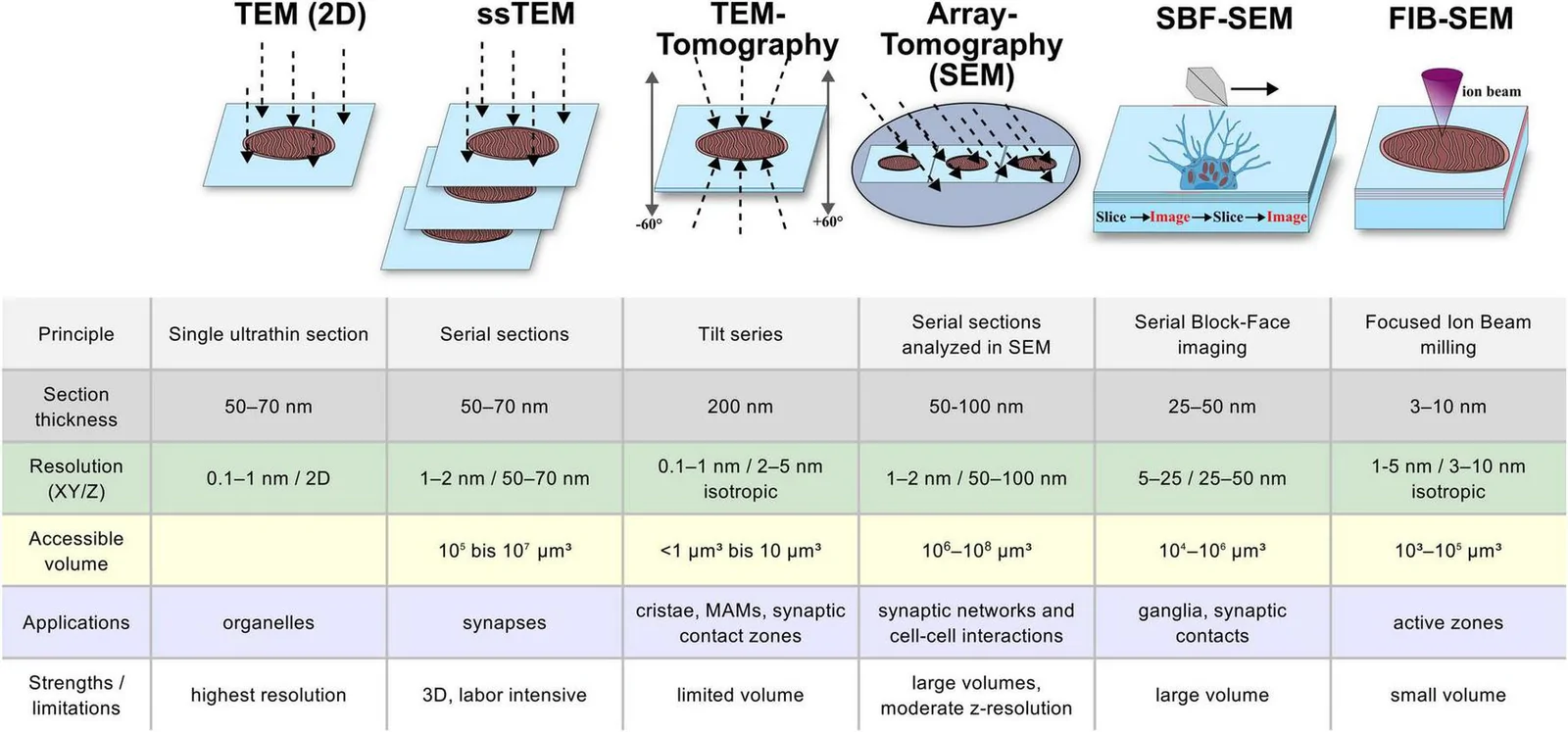
Overview of electron microscopy (EM) approaches for ultrastructural analysis of the enteric nervous system (ENS). Conventional transmission electron microscopy (TEM), serial-section TEM (ssTEM), TEM-tomography, array tomography, serial block-face scanning electron microscopy (SBF-SEM) and focused ion beam scanning electron microscopy (FIB-SEM) are compared in terms of their applications and capabilities for imaging cellular ultrastructure and neuronal networks.

Despite these optimized preparation and preservation methods, ultrastructural analysis remains essentially limited to two-dimensional TEM data. Although it has been the standard method for ultrastructural analysis of the ENS for decades (Baumgarten et al., 1970; Gabella, 1972; Cook and Burnstock, 1976a; Wilson et al., 1981), it is limited in quantitative morphological analysis, such as that of mitochondrial shape, due to its two-dimensional nature (Mannella, 2006; Peddie et al., 2022). Complex three-dimensional structural changes cannot be adequately captured using these methods (Heinen-Weiler et al., 2021). Against this backdrop, three-dimensional EM techniques are increasingly being used as a methodological extension and are considered necessary (Peddie et al., 2022) to map the spatial organization of the ENS more consistently (Figure 5). The oldest method is manual serial-section reconstruction (Serial Section TEM, ssTEM) (Birch-Andersen, 1955; Schneider et al., 2021). Serial-section TEM enables three-dimensional reconstruction from consecutive ultrathin sections but remains limited by high manual workload and reconstruction artifacts (Briggman and Denk, 2006; Shi and Zhu, 2026). However, due to the high level of manual effort required, ssTEM remains limited to small volumes ranging from individual cells to small neural networks (Briggman and Denk, 2006). However, section loss, distortions, alignment errors, and interpolation during reconstruction limit accuracy in large volumes. A first step toward further development is represented by electron tomography methods (TEM tomography), in which a single section (typical section thickness 200 nm) is imaged from different tilt angles and computationally reconstructed in three-dimensions (Lucic et al., 2005; Gan and Jensen, 2012). TEM tomography enables high-resolution three-dimensional reconstruction of subcellular structures but remains limited to small volumes, making it suitable for mitochondrial cristae, synaptic contact zones, and subcellular organization in the ENS (Perkins et al., 1997; Lucic et al., 2005; Fernandez-Busnadiego et al., 2010; Gan and Jensen, 2012). In addition, array tomography enables the serial analysis of ultrathin sections mounted on substrates like silicon wafers, which are automatically imaged and reconstructed (Hayworth et al., 2014; Peddie and Collinson, 2014). This allows for the analysis of larger volumes and could be used to study synaptic networks and cell-cell interactions in the ENS (Briggman and Bock, 2012; Hayworth et al., 2014; Kasthuri et al., 2015). Serial Block-Face-Scanning Electron Microscopy (SBF-SEM) enables automated serial imaging and allows analysis of significantly lager tissue volumes compared with conventional approaches (Denk and Horstmann, 2004; Peddie and Collinson, 2014; Schneider et al., 2021). In the CNS, this approach has enabled three-dimensional visualization of synaptic organization (Rollenhagen et al., 2020). In the ENS, this methodology could open the possibility of reconstructing entire enteric ganglia, including neuronal and glial networks, in three dimensions and analyzing their spatial organization within the intestinal wall. Focused Ion Beam-SEM (FIB-SEM) represents the highest-resolution automated 3D-EM technique within this spectrum. Tissue is ablated layer by layer using a FIB and simultaneously imaged by SEM (Heymann et al., 2006; Xu et al., 2017). This achieves a nearly isotropic resolution in the range of ∼3–10 nm, though with a significantly limited analyzable volume in the range of ∼10^3^–10^5^ μm^3^ (Knott et al., 2008; Peddie and Collinson, 2014; Xu et al., 2017). In the ENS, this method could be particularly suitable for the detailed analysis of subcellular structures such as mitochondrial networks, synaptic varicosities, and neuron-glial contact sites (Peddie et al., 2022). At the same time, the targeted identification of specific structures within the complex tissue context of the ENS poses a methodological challenge, as spatial orientation within the volume limits the localization of specific anatomical regions (Loginov et al., 2022). Therefore, a combination with correlative methods such as CLEM or upstream 3D light microscopy is often necessary to enable targeted navigation within the tissue (Iwasaki et al., 2022; Loginov et al., 2022). These approaches represent a methodological spectrum, with ssTEM enabling cellular reconstructions, SBF-SEM providing larger volume analysis, and FIB-SEM resolving subcellular organization (Peddie and Collinson, 2014; Schneider et al., 2021; Peddie et al., 2022). In the context of the ENS, these state-of-the-art 3D techniques could in the future extend our ability to investigate the ultrastructural organization of enteric neuronal and glial networks, complementing advanced three-dimensional optical imaging approaches that already allow analyses of tissue architecture and neuronal network organization. Furthermore, these approaches could enable investigation of enteric neuronal and glial network organization, synaptic architecture, mitochondrial morphology, and structural correlates of PD-associated processes such as α-synuclein pathology.

## 6 Ultrastructural analyses in ENS research: a subcellular level that has remained largely unexplored

Despite their considerable potential, the application of advanced 3D-EM approaches in ENS research remains limited. This is mainly related to their high technical complexity, extensive requirements for data acquisition and analysis, and the specialized expertise required for ultrastructural reconstruction. In addition, ultrastructural studies of the ENS, particularly those involving human patient material, are inherently constrained by the limited availability of suitable samples. Human intestinal tissue obtained from well-characterized patient cohorts is difficult to access, resulting in relatively small sample sizes (Iruzubieta et al., 2022). Consequently, it is not surprising that the majority of available ultrastructural studies of the ENS are still based on classical TEM analyses, many of which were performed in the 1970s and 1980s. Gabella (1972), Baumgarten et al. (1970) are considered pioneers of this electron microscopic research. Gabella (1972) provided what is arguably the most detailed ultrastructural description. He described enteric ganglia as a densely packed network of neuronal and glial structures without interposed connective tissue, resulting in structural parallels to the CNS (Gabella, 1972). Ultrastructurally, enteric glial cells are characterized by a filament-rich cytoplasm and close contacts with neuronal cells (Gabella, 1972; Cook and Burnstock, 1976b; Wilson et al., 1981). In contrast to glial cells of the peripheral nervous system (PNS), however, they do not form myelin sheaths and only partially surround the axons (Baumgarten et al., 1970; Gabella, 1972). These early observations already provided evidence of a structurally and functionally distinct population of glial cells that differs from both the CNS and the PNS. Enteric neurons also exhibit characteristic axonal varicosities that contain numerous vesicles and function as central sites of neurotransmitter release (Baumgarten et al., 1970; Wilson et al., 1981). Baumgarten et al. (1970) focused on these structures and described three different types of varicosities that can be distinguished based on their vesicle composition. In addition to small, clear vesicles, larger, dense vesicles were also identified that are associated with various transmitter systems (Baumgarten et al., 1970). These findings underscore the functional diversity and complex organization of signal transmission in the ENS. In addition, Cook and Burnstock (1976a) classified enteric neurons and axon profiles based on ultrastructural features and attempted to correlate them with one another.

Although this pioneering work was conducted several decades ago, there are few recent studies that employ comparable ultrastructural approaches to investigate the ENS. More recent studies are largely limited to specific research questions or model organisms. Using modern scanning TEM, all connections between the ENS and the CNS, as well as the ENS itself, were visualized in Drosophila larvae (Schoofs et al., 2024). This technique, particularly when combined with other methods, opens new possibilities for gaining deeper insight into the complex communication of the gut-brain axis and for developing an understanding of potential anatomical pathways of α-synuclein pathology. Baker et al. (2019) investigated the development of the myenteric plexus in zebrafish and, using TEM, were able to demonstrate the close interaction between neurons and glial cells during the development of the ENS. Furthermore, Zhang et al. (2024) investigated the ultrastructural morphology of enteric glial cells in the cecum and largely confirmed the characteristics previously described by Gabella (1972). The group led by Nakanishi et al. (2020) used SBF-SEM to visualize the innervation of the ileal mucosa in three dimensions. This methodology also holds great potential for application to disease models, as it allows for detailed examination of levels of the ENS that have rarely been analyzed to date. Furthermore, it is precisely here that the neurons are located that are in close spatial proximity to potential luminal pathogens. Nevertheless, these data are limited to anatomical descriptions in the healthy model organism. Particularly given that EM studies are predominantly used in a complementary manner and serve primarily for qualitative characterization, electron microscopic images generally have a supplementary function and are not part of direct quantitative analyses. Böttner et al. (2015), for example, used SEM to detect axonal varicosities in the myenteric plexus after characterizing them via fluorescence microscopy as sites of α-synuclein expression. However, further ultrastructural analyses, such as CLEM, which would have enabled the ultrastructural localization of the target structure, were not performed. Another research group investigated the effects of electroacupuncture on PD in the ENS (Song et al., 2022). The ultrastructural analysis of unmyelinated nerve fibers revealed a significant increase in the density of these fibers (Song et al., 2022). This finding led to the conclusion that this might be a possible cause of the improvement in constipation in PD. Here, the focus was exclusively on the nerve fibers. There were no descriptions of organelles or even cell morphology. Particularly in the context of PD, there is an urgent need to further deepen such investigations, as mitochondrial dysfunction represents a central pathomechanism of the disease. Given the described increased nerve fiber density, this gains additional relevance, as structural changes at the axonal level are potentially linked to subcellular pathological processes. For multiple sclerosis (MS), EM analyses have already provided corresponding mechanistic insights. In a mouse model of experimental autoimmune encephalomyelitis, for example, Wunsch et al. (2017) demonstrated the involvement of the ENS in MS. They quantitatively assessed the number of degenerating axons, axon density, and the proportion of mitochondrial area relative to axon area (Wunsch et al., 2017). In addition to axonal degeneration, mitochondrial swelling was also detected, suggesting possible mechanisms by which mitochondria contribute to the degeneration (Wunsch et al., 2017).

This highlights the need to consistently apply these promising methodological approaches to other neurodegenerative disease models. This is particularly relevant given the currently limited ultrastructural data set, which only reaches its full potential in conjunction with light-microscopic and functional studies (Table 2).

**TABLE 2:** Ultrastructural findings in the enteric nervous system.

References	Species	Disease	Method	Main finding
Baumgarten et al., 1970	Human, rhesus monkey, guinea pig	Healthy	TEM	Described three classes of neuronal processes in myenteric ganglia based on vesicle content: agranular vesicles, small granular vesicles, and large dense-core vesicles.
Gabella, 1972	Guinea pig ileum	Healthy	TEM	Myenteric ganglia consisted of tightly packed neurons and glial cells without intervening connective tissue; glial cells contained abundant filaments and incompletely ensheathed axons.
Cook and Burnstock, 1976a	Guinea pig	Healthy	TEM	Classified neuronal elements and axon profiles of Auerbach’s plexus according to ultrastructural characteristics and vesicle populations.
Cook and Burnstock, 1976b	Guinea pig	Healthy	TEM	Characterized non-neuronal elements including enteric glia and fibroblast-like cells; glia lacked myelin and contained numerous filaments.
Wilson et al., 1981	Guinea pig ileum	Healthy	TEM	Described submucosal ganglia composed of neurons, Schwann cells and neuropil; identified synaptic contacts and vesicle-containing varicosities.
Böttner et al., 2015	Human, rat	Parkinson’s disease	SEM + immunofluorescence	α-Synuclein localized to synaptic vesicle-associated structures and varicosities in the myenteric plexus.
Wunsch et al., 2017	Mouse (EAE)	Multiple sclerosis	TEM	Increased numbers of degenerating axons and swollen mitochondria in enteric nerve fibers; reduced axonal density indicated neurodegeneration in the ENS.
Baker et al., 2019	Zebrafish larva	Development	TEM	Revealed formation of a neuropil pattern and close association between enteric neurons and glial processes during ENS maturation.
Nakanishi et al., 2020	Rat	Healthy	SBF-SEM	Reconstructed three-dimensional innervation of ileal mucosa; showed direct contacts of nerve fibers with epithelial and stromal cells.
Iruzubieta et al., 2022	Human	Healthy	TEM + ssTEM	TEM demonstrated the ultrastructural architecture of human myenteric ganglia and identified primary cilia in enteric glial cells and interstitial cells of Cajal, supporting the presence of neurogenic niche features in the adult human ENS
Song et al., 2022	Rat	Parkinson’s disease model	TEM	Parkinsonian rats showed reduced density and degeneration of unmyelinated nerve fibers; electroacupuncture increased fiber density and restored ultrastructural integrity.
Schoofs et al., 2024	*Drosophila* larva	Healthy	Scanning TEM (connectomics)	Generated a complete gut–brain connectome and reconstructed serotonergic circuits controlling swallowing.
Zhang et al., 2024	Chicken cecum	Healthy	TEM	Enteric glial cells exhibited irregular nuclei, filament-rich cytoplasm and close associations with neuronal elements, resembling mammalian enteric glia.

## 7 Conclusion and outlook

However, the temporal and spatial sequence of pathological events remains a matter of ongoing debate, as PD is increasingly recognized as a heterogeneous disorder with different possible sites of disease initiation (Borghammer and Van Den Berge, 2019; Horsager et al., 2020). Numerous experimental and clinical studies provide evidence for early involvement of the ENS in PD through the detection of misfolded α-synuclein aggregates in the ENS, both in animal models and in patient biopsies (Wakabayashi et al., 1988; Braak et al., 2006; Lebouvier et al., 2008; Drolet et al., 2009; Lebouvier et al., 2010; Shannon et al., 2012; Wang et al., 2012; Ohlsson and Englund, 2019). Propagation along the vagus nerve also appears plausible, as a lower prevalence of PD was observed in a specific population of patients who underwent truncal vagotomy compared to the general population (Svensson et al., 2015). Furthermore, this was confirmed in an animal model following the injection of α-synuclein PFFs into the muscular layer of the small intestine, where central pathologies and a loss of dopaminergic neurons were observed, supporting the theory of prion-like spread (Kim et al., 2019). Although numerous studies support early involvement of the ENS, there are also conflicting data that call into question the generalizability of the hypothesis. In some studies, no consistent presence of α-synuclein in the ENS could be detected in early stages of the disease (Lebouvier et al., 2011; Devos et al., 2013); furthermore, not all patients exhibit the spread pattern described by Kalaitzakis et al. (2008), Parkkinen et al. (2008). The pathogenesis of PD is thus more heterogeneous (e.g., body-first, brain-first) than originally assumed, and it remains unclear whether the ENS actually represents the primary site of origin of the disease or is rather an early-affected component of a systemic neurodegenerative process (Borghammer and Van Den Berge, 2019; Horsager et al., 2020). Ultrastructural approaches may help to address these open questions by identifying early subcellular alterations within the ENS, such as mitochondrial dysfunction, organelle remodeling, synaptic changes, or pathological α-synuclein-associated structures. Such analyses may provide insights into whether ultrastructural alterations represent primary disease-associated events within the ENS or rather secondary consequences of a broader neurodegenerative process. The ongoing controversies surrounding the Braak et al. (2019) hypothesis and alternative models of PD pathogenesis underscore the need for ultrastructural studies of the ENS. While α-synuclein deposits and functional changes have already been described on multiple occasions, data systematically characterizing the underlying subcellular mechanisms are still lacking.

Ultrastructural research on the ENS is therefore not merely a methodological necessity but could make a decisive contribution to understanding early neurodegenerative processes and identifying new diagnostic and therapeutic targets. Against this backdrop, however, it becomes clear that the currently available methodological approaches fall short of meeting this need. Despite the extremely diverse range of methods for ultrastructural analysis, the ENS remains largely unexplored, particularly in the context of pathological changes. Most current studies are based on immunohistochemical and fluorescence-based methods which provide valuable information on protein localization, neuronal and glial morphology, tissue architecture and functional organization of enteric networks. Nevertheless, these approaches remain limited in their ability to resolve ultrastructural alterations at nanometer resolution, particularly with respect to intracellular organelles, synaptic architecture and membrane organization. Furthermore, the overall picture is heterogeneous, as functional and structural findings are often inconsistent and depend heavily on the cell population, intestinal region, and methodological approach. This discrepancy highlights the fundamental limitation of conventional light microscopy techniques, whose resolution fails to adequately capture the underlying ultrastructure. In particular, the organization of subcellular structures remains largely inaccessible at the organelles such as mitochondria and MAMs, as well as the spatial distribution of pathological α-synuclein aggregates, can only be captured fragmentarily at this level. The majority of available studies are descriptive in nature and focus on the myenteric plexus, while the submucosal plexus is investigated much less frequently (Baumgarten et al., 1970; Gabella, 1972; Cook and Burnstock, 1976a; Böttner et al., 2015; Wunsch et al., 2017; Baker et al., 2019; Song et al., 2022). Even at the subcellular level, analysis is predominantly qualitative; organelles such as mitochondria or the ER are described, but rarely systematically quantified or placed in functional contexts. Furthermore, most ultrastructural studies are conducted on tissue sections. Ultrastructural investigations of isolated enteric neurons or glial cells are largely absent. Despite the extensive ultrastructural descriptions of glial cells, there is a lack of in-depth studies focusing on this cell population, particularly in the context of pathological conditions. Emerging evidence suggests that enteric glial cells actively contribute to PD-associated neuroinflammation rather than merely responding to neuronal injury. In patients with PD, enteric glial cells exhibit features of reactive gliosis, including increased expression of glial markers such as GFAP and S100β, accompanied by elevated levels of pro-inflammatory mediators such as IL-1β, IL-6, and TNF-α (Clairembault et al., 2014; Benvenuti et al., 2020; Montalbán-Rodríguez et al., 2024). Through activation of immune-related signaling pathways, including Toll-like receptor (TLR)-2 and TLR4 signaling, reactive enteric glia has been proposed to contribute to local inflammatory responses, altered intestinal barrier integrity, and the establishment of pro-inflammatory microenvironment (Benvenuti et al., 2020). A bidirectional interaction between enteric glial activation and α-synuclein pathology has been proposed, although the underlying mechanisms remain incompletely understood (Clairembault et al., 2015). However, whether these functional alterations are accompanied by characteristic ultrastructural changes remains largely unknown. However, whether these functional alterations are accompanied by characteristic ultrastructural changes remains largely unknown. Integrating ultrastructural approaches with functional models, including cultured enteric glial cells and neurons, may help to be better understand their contribution to neurodegenerative processes. Immunogold labeling could be used to simultaneously identify potential modulators of inflammatory and degenerative mechanisms. Apart from this, 3D applications have so far been used only very sporadically, even though they would be essential for a spatially accurate characterization of subcellular organization and should be part of the methodological standard in both qualitative and quantitative analysis. Studies of the CNS have been pioneers in this regard, while many details of the ENS remain hidden. These methodological limitations highlight the fact that an in-depth analysis at the ultrastructural level is indispensable. There is a lack of comprehensive studies that systematically link subcellular structures to disease-relevant changes in the ENS. In PD, in particular, greater focus should be placed on mitochondrial changes, as these play a central role in the context of oxidative stress and disrupted energy homeostasis.

In addition, increasing attention should be directed toward the interaction between the gut microbiome and the ultrastructural organization of ENS cells. Despite the growing evidence that microbial metabolites, inflammatory mediators and alterations in intestinal barrier integrity can influence neuronal homeostasis, it remains still unknown whether microbiome-associated changes are reflected at the ultrastructural level (Fung et al., 2017; Cryan et al., 2019; Morais et al., 2021). Elucidating these relationships could provide important insights into how environmental factors contribute to early neurodegenerative processes and may help explain the heterogeneity observed in PD. Despite the considerable potential of modern EM and 3D imaging approaches, the ENS has so far been only marginally considered in ultrastructural research. A major reason for this lies in the technically demanding and time-consuming nature of sample preparation. While conventional light microscopy analysis can be performed within minutes to hours, classical EM requires a multi-day, often approximately 1-week preparation process, including fixation, dehydration, embedding, and ultrathin sectioning. In addition, the subsequent steps of image acquisition and, in particular, software-assisted post-processing and 3D reconstruction of ultrastructural data are extremely time-consuming and require considerable manual effort, specialized analysis software, and a high level of methodological expertise. As a consequence of these requirements, many ultrastructural investigations are performed on relatively small sample sizes. This applies to both animal models and, in particular, human patient material, as well-characterized ENS samples are difficult to obtain. Due to the limited availability of human ENS samples, many studies rely on animal models, which may not fully reflect the complexity and heterogeneity of human Parkinson’s disease (Song et al., 2022; Garcia-Caballero et al., 2026). Furthermore, many existing studies are still primarily based on conventional TEM and provide mainly qualitative descriptions. Although ssTEM has enabled three-dimensional reconstructions for decades, its application remains limited by the substantial manual workload. Each individual section must be precisely prepared, transferred, imaged, and subsequently aligned. Section loss, local distortions, and the need for interpolation during reconstruction can further compromise the accuracy of larger 3D datasets. Modern automated approaches such as SBF-SEM can overcome some of these limitations, as image acquisition is performed directly within the system and does not require individual section transfer. This enables more efficient acquisition of larger volumes; however, subsequent segmentation and reconstruction of complex biological structures remain major analytical challenges. These limitations highlight the need for approaches that enable more efficient and quantitative analysis of ultrastructural datasets. In particular, artificial intelligence-based image analysis offers new opportunities for the automated and unbiased quantification of ultrastructural features, including mitochondrial morphology, synaptic organization, and organelle interactions. The use of such techniques has the potential to significantly increase throughput and reproducibility while enabling the analysis of large and complex datasets. In addition, three-dimensional ultrastructural approaches such as FIB-SEM technology were originally developed outside the life sciences, particularly in the context of semiconductor and materials research, and have only in recent years been increasingly transferred to biological and medical applications. Accordingly, their use in neuroscience has initially been strongly focused on the CNS. This is partly because the ENS was long not considered a primary site of origin or early manifestation of neurodegenerative diseases such as PD. Consequently, research efforts have predominantly focused on CNS structures, particularly in the context of investigating potential disease origins in the brain.

However, despite these methodological advances, several technical and conceptual gaps remain that still limit comprehensive ultrastructural and molecular characterization of the ENS. In particular, state-of-the-art preservation techniques such as HPF, which are essential for near-native structural preservation, are still not routinely applied in many ENS studies due to their technical complexity and limited accessibility. As a consequence, ultrastructural analyses are often still based on conventionally fixed tissue, which may not fully preserve labile subcellular structures or dynamic organelle interactions. This methodological gap is further compounded by the fact that EM, despite its high spatial resolution, remains largely decoupled from molecular readouts, thereby limiting the ability to directly relate ultrastructural organization to underlying molecular states. Bridging this gap requires integrative approaches that combine structural and molecular information within the same cellular context. Moreover, combining EM with emerging spatial omics approaches could bridge the gap between molecular signatures and ultrastructural phenotypes by directly linking transcriptomic and proteomic information to specific cellular compartments. In addition, cryogenic EM and cryo-electron tomography may further enable near-native preservation of cellular structures and allow three-dimensional visualization of pathological protein aggregates and organelle networks at unprecedented resolution.

Taken together, these technical and conceptual factors explain why the ENS remains significantly underrepresented at the ultrastructural level compared to the CNS. At the same time, recent methodological advances and the increasing automation of image acquisition and analysis suggest that EM and 3D reconstruction techniques will become central components of future ENS research. This may ultimately enable systematic analyses of subcellular structures in the context of neurodegenerative diseases and lead to the identification of novel ultrastructural biomarkers.
